# Sex and Gender Disparities in Melanoma

**DOI:** 10.3390/cancers12071819

**Published:** 2020-07-07

**Authors:** Maria Bellenghi, Rossella Puglisi, Giada Pontecorvi, Alessandra De Feo, Alessandra Carè, Gianfranco Mattia

**Affiliations:** 1 Center for Gender-specific Medicine, Istituto Superiore di Sanità, 00161 Rome, Italy; maria.bellenghi@iss.it (M.B.); rossella.puglisi@iss.it (R.P.); giada.pontecorvi@iss.it (G.P.); gianfranco.mattia@iss.it (G.M.); 2 Laboratory of Experimental Oncology, IRCCS Istituto Ortopedico Rizzoli, 40136 Bologna, Italy; alessandra.defeo@ior.it

**Keywords:** melanoma, sex/gender, sex-hormones, immunity, microRNAs, immunotherapy

## Abstract

Worldwide, the total incidence of cutaneous melanoma is higher in men than in women, with some differences related to ethnicity and age and, above all, sex and gender. Differences exist in respect to the anatomic localization of melanoma, in that it is more frequent on the trunk in men and on the lower limbs in women. A debated issue is if—and to what extent—melanoma development can be attributed to gender-specific behaviors or to biologically intrinsic differences. In the search for factors responsible for the divergences, a pivotal role of sex hormones has been observed, although conflicting results indicate the involvement of other mechanisms. The presence on the X chromosome of numerous miRNAs and coding genes playing immunological roles represents another important factor, whose relevance can be even increased by the incomplete X chromosome random inactivation. Considering the known advantages of the female immune system, a different cancer immune surveillance efficacy was suggested to explain some sex disparities. Indeed, the complexity of this picture emerged when the recently developed immunotherapies unexpectedly showed better improvements in men than in women. Altogether, these data support the necessity of further studies, which consider enrolling a balanced number of men and women in clinical trials to better understand the differences and obtain actual gender-equitable healthcare.

## 1. Introduction

Melanoma is the most aggressive type of skin cancer, at present accounting for 1% of total cancer deaths in Italy. For a long time, only the surgical resection of early lesions was associated with long-term survival in more than 90% of patients, whereas advanced melanomas were mostly incurable. Although in the last decades a steadily increasing incidence of cutaneous melanoma was observed worldwide, an important 18% decrease in mortality was recently associated with improved knowledge of biological data and the introduction of novel therapeutic approaches, melanoma reduction being the highest among the other major cancers [1]. 

The incidence and mortality rate of the disease differ widely across the globe depending on the country of residence, ethnicity, and socioeconomic conditions and, chiefly, access to early detection and primary care [2]. It is also of note that incidence gradually decreases going from Northern to Southern Italy [1].

An additional key variable in melanoma is gender, in that a female advantage has been generally reported. Among the younger Italian population (under 50 years old), melanoma represents the 2nd most frequent tumor in men and the 3rd in women, the risk of developing this type of cancer during the life course being 1:66 and 1:85, respectively. In both sexes, the incidence is rising, with a 4.4% increase in men and a 3.1% increase in women per year. In 2019, 12,300 new cases were expected, with little prevalence in males [1].The mechanisms underlying gender disparity in melanoma development are not clear enough. Lifestyles play a role, with ultraviolet exposure representing an important risk factor, as women are more interested in sun exposure and tanning [3]. Conversely, males are generally less likely to engage in preventive behaviors [4] or to self-detect their melanomas [5]. Indeed, a different readiness of detection might be associated with the gender body-site distribution being primary melanomas more truncal in males and localized on the lower extremities in females. Thus, also an earlier diagnosis can partly explain the better survival rate of women.

As for the histological features, although thicker and ulcerated tumors were more frequently observed in men, these elements do not seem responsible for the unfavorable prognosis compared to women [6]. A large part of the female survival advantage could be explained with lower dissemination, resulting in a reduction in both lymph nodes and distant metastases when compared with males [7], and even after spreading to a visceral organ, a better prognosis seems to persist for women [8].

Looking for genetic differences, it is important to note that in women, the random—and sometimes incomplete—inactivation of one X chromosome in each single cell leads to mosaicism, and in turn, to the advantages associated with female genetic heterogeneity [9]. 

A significantly higher number of missense mutations was found among men with a mutational load ratio Men-to-Women of 1.85. Although the number of mutations is lower in melanoma female patients, their presence appears more relevant for increasing the overall survival, suggesting the functional pressure of the more efficient female immune system [10]. Furthermore, a study conducted in a Hispanic population identified several Single Nucleotide Polymorphisms (SNPs) differently associated with pigmentation, sun tolerance and melanoma risk in a sex-related manner [11]. 

Sex hormones play a fundamental role, as several studies demonstrated the association of estrogen and estrogen receptor expressions with melanoma survival in women. Notably, the female immune system is more efficient than the male one, and women mount both innate and adaptive immune responses stronger than men do. This higher effectiveness on the one hand is an advantage against infectious diseases and cancers, while on the other, it makes women more prone to autoimmune diseases [12]. 

Here we report the main disparities between men and women in an attempt to understand the sex- or gender-related effects on melanoma development, progression and response to therapy. We also speculate on the regulatory function possible played by miRNA that affects sex differences in melanoma pathogenesis at hormonal and immune levels. 

## 2. Sex Steroid Hormone Receptors in Melanoma

Sex hormones belong to the steroid hormone family, mainly synthesized by the adrenal cortex and gonads, and in minor part by several peripheral tissues, such as the skin. In fact, starting from blood precursors or mobilizing cholesterol from cellular stores, the skin is able to produce several biologically active steroids, such as estrogens, testosterone (T) and dihydrotestosterone (DHT) [13]. Of course, the endocrine function of the skin becomes of particular relevance in men and in postmenopausal women, when almost all estrogens are made through the extraglandular conversion of androgens into estrogens. Estrogens exert their biological effects by binding to and activating two members of the nuclear steroid receptor superfamily, the estrogen receptor α (ERα) and β (ERβ), as well as the more recently discovered G protein-coupled estrogen receptor (GPER). While both genomic and non-genomic pathways have been described for the signaling activities of ERs, GPER is believed to mediate a rapid and non-genomic response upon hormone binding. Estrogens regulate the growth and differentiation of normal and several neoplastic tissues (such as breast, ovarian and endometrial tumors). Indeed, in cancer growth, ERs exert the opposite effects, ERα being pro- and ERβ anti-proliferative [14]. It is believed that cutaneous ER levels are generally higher in women than in men [15,16]. Some immunohistochemical analyses indicated that in melanocytic nevi and malignant melanoma cells, ERβ was present but ERα was not [17,18], even if both ERα and ERβ mRNAs were found in several melanocytic lesions [19]. Indeed, the presence or not of ERα either in primary or in metastatic melanoma is an unresolved issue. Downmodulation of this receptor has been shown to be under epigenetic control and appears to be directly proportional to disease progression [20]. In this perspective, the detection of hypermethylated ERα in melanoma patient sera was proposed as a predictive marker of bio-chemo-therapy response, thus becoming a negative prognostic factor [20]. Noteworthy, ERα is also able to synergize with the insulin-like growth factor 1 receptor (IGF1R) in response to 17β-estradiol (E2) and IGF1 stimuli, as showed in the MCF7 breast cancer cell line [21]. A recent work on melanoma shows the presence of one SNP in IGF1 and another in IGF1R, likely associated with increased risk or protective effect, respectively, especially in men. In line with this, the possible role of ESR1 SNPs in melanoma requires further investigation [22]. Conversely ERβ expression is inversely correlated with Breslow thickness, the most important and independent predictive marker of melanoma [16]. According to the survival advantage of female melanoma patients, men show significantly lower levels of ERβ in both melanoma and healthy tissues [16]. A more recent study on melanoma survival ratios from the Human Protein Atlas and The Cancer Genome Atlas Genomic Data Commons (GDC) showed that while low ERβ expression was associated with shortened relapse-free survival (RFS), ERα and GPER were not [23]. Concerning in vitro studies, several melanoma cell lines express ERβ, irrespective of genetic background [24]. GPER is also expressed in melanoma [25] its co-expression with ERβ being associated with better outcomes, especially in pregnancy-associated melanoma [26]. The nuclear receptor superfamily also includes the androgen receptor (AR), consisting of α and β isoforms encoded by a gene located on the X chromosome [27]. AR respond to androgenic hormones by using the same genomic and non-genomic pathway of ERs and evoking similar effects [28]. Although less studied than ERs, the expression of AR was assessed in several melanoma cell lines and in human metastatic specimens, where high receptor levels were detected [29]. Concerning progesterone (P), to date, different receptors (PRs) that are able to activate both genomic and non-genomic pathways have been identified [27]. Since melanocytic lesions seem to change during pregnancy, several attempts have been made to associate PR expression to melanoma course in pregnant and in non-pregnant women, without reaching definitive results [30].

## 3. Female Hormone Activity

A large body of evidence supports the beneficial role played by estrogens against melanoma progression. Epidemiological studies have pointed out how menarche and menopause with consequent changes in the endogenous estrogen exposure can influence melanoma risk [31]. However, the persistence of female benefit in older postmenopausal women is an unresolved issue due to conflicting results published over time [32,33,34]. Controversial data were reported for exogenous estrogens, such as oral contraceptive [35] and hormone-replacement therapy (HRT). Concerning the latter point, some European cohort studies recently showed an increased risk in melanoma associated with use of HRT [36,37,38,39,40], whereas others did not [31,41]. Likewise, the attempt to associate pregnancy with melanoma outcomes did not reach conclusive results [42,43].

**Figure 1:**
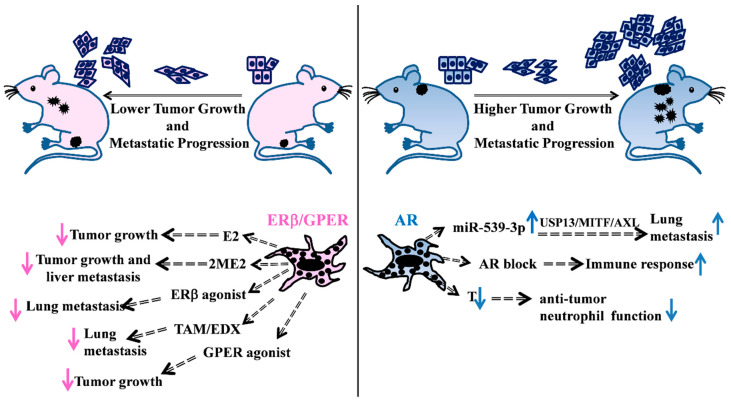
Schematic overview of some representative in vivo studies supporting the hormonal involvement in melanoma disease. (**Left**) Evidences of female hormonal treatments in in vivo mice models. E2 treatment of castrated mice s.c. injected with ER positive melanoma cells reduces tumor growth [47]. 2ME2 treatment of SCID mice, previously intrasplenically injected with melanoma cells, decreases primary tumor growth and liver metastasis number [49]. Treatment of melanoma cells with GPER agonist affects tumor growth in host mice improving response to immunotherapy [54]. Administration of the ERβ agonist LY500307 in female mice, previously i.v. injected with melanoma cells, decreases lung nodules [55]. Oral administration of TAM [56] and EDX [57] exert inhibitory effects on tumor metastatization into the mouse lungs. (**Right**) AR action is responsible for the increased number of lung metastases through the miR-539-3p/USP13/MITF/AXL axis [58], whereas AR blockade mediates an increase in the immune response [59]. T loss in castrated male mice causes a decrease in the anti-tumor neutrophil function [60]. E2: 17β-estradiol; 2-ME2: 2-methoxyestradiol; TAM: Tamoxifen; EDX: endoxifen; USP13: ubiquitin specific peptidase 13; MITF: microphthalmia-associated transcription factor; AXL: receptor tyrosine kinase AXL; T: testosterone. s.c. subcutaneous, i.v. intravenous.

In melanoma cell lines, in vitro response to 17β-estradiol (E2) treatment produced different effects on either proliferation or invasion ability [44,45,46]. In other reports, only in vivo mouse models (Figure 1, left) were able to show the estrogenic capability to contrast disease progression. As demonstrated in athymic nude mice, the same melanoma cell line unresponsive to estradiol treatment in vitro displayed significantly reduced growth in castrated mice treated with 17-β estradiol pellets, suggesting other possible indirect hormone actions [47]. A growing interest in the use of 2-methoxyestradiol (2ME2), a non-toxic endogenous metabolite of E2, highlighted its ability to block the human melanoma cell-cycle, inducing apoptosis both in vitro [48] and in male severe combined immunodeficient (SCID) mouse models [49]. Sex-related differences in metastasis formation were principally observed in the liver, the organ mainly active in estrogen conversion into 2ME2 [50]. This was in perfect agreement with results previously observed in SCID mice where, as a consequence of intrasplenic injection of ER positive human melanoma cells, a greater number of metastases was observed in the male liver than in that of the female [51]. Indeed, the estrous cycle of female mice could also affect the capability of B16 melanoma cells to metastasize in different organs [52]. More recently, the antiproliferative and cytotoxic effects of 2ME2 were shown in melanoma cells with different genetic backgrounds, as well as in the counterparts resistant to either BRAFi (v-raf murine sarcoma viral oncogene homolog B1) or the BRAFi+MEKi (Mitogen-Activated Protein Kinase Kinase) combination [53]. 

Besides hormones and their metabolites, several natural and synthetic compounds with different affinities to ERs and divergent activities, i.e., agonist/antagonist, were tested in vitro and in vivo. It is worth remembering the ERβ agonist diarylpropionitrile (DPN) efficacy in inhibiting NRAS (Neuroblastoma RAS Viral Oncogene Homolog)-mutated melanoma cell proliferation [24], as well as many specific ERβ agonists of natural origin able to exert an antitumor function in different human melanoma cell lines in vitro and in vivo [61]. Recently, the synthetic non-steroidal estrogen and selective ERβ agonist LY500307 showed the capability to suppress melanoma lung metastasis in the B16 murine melanoma in vivo model by up-regulating innate immunity in a tumor microenvironment [55]. Tamoxifen (TAM), widely used for the treatment of both early and advanced breast cancer, belongs to the selective estrogen receptor modulator (SERM) class of drugs acting on the ERs [62]. TAM’s effectiveness in melanoma treatment is due to the discordant data derived from both in vitro and in vivo studies. Numerous results have described SERM-dependent inhibition of melanoma cell proliferation, suggesting the possible involvement of IGF1R inactivation [63] as well as the reduction of invasion and metastasis through the inhibition of protein kinase C (PKC) downstream pathways [56]. Unfortunately, the activity of TAM against melanoma progression is extremely poor in vivo [64], unless combined treatments with chemotherapy are considered. In the latter condition, a better response to chemotherapy treatment was observed in advanced melanoma, with female patients being more likely to respond, albeit with increased toxicity and a doubtful survival advantage [65]. TAM’s clinical efficacy, at least in part, depends on its metabolization in the liver, resulting in variable concentrations of active metabolites in the patient plasma. Therefore, to induce melanoma cell death in vitro, endoxifen (EDX), a metabolite of TAM that is safer and has more cytostatic activity than the parent drug has been used [66]. Furthermore, EDX orally administered in mice for four weeks reduced lung metastatic nodules without any side effects [57]. Another reason for different epidemiological evidence in TAM’s effectiveness could lie in its possible different and tissue-related effects on the two estrogen receptors, similarly to E2 [67,68]. The recent GPER inclusion among TAM-responsive receptors in melanoma further complicated the scenario [25]. Several studies described the ability of GPER to react with E2 and its specific agonist G-1 to evoke a suppressive action against melanoma progression [25,69,70]. In addition, GPER’s ability to make melanoma more vulnerable to immune-mediated eradication upon hormone treatment was reported [54]. Several in vitro studies showed the concentration-dependent progesterone activity on several human melanoma cell line growth, adhesion and migration abilities [71,72,73,74,75]. According to these in vitro results, repeated pregnancies inhibited the development of genetically defined BRAF-driven human melanocytic xenografts when compared with non-pregnant females [54].

## 4. Male Hormone Activity

A large body of evidence supported AR involvement in melanoma incidence and progression, although a clear correlation between AR expression in melanoma and bad prognosis is lacking. Many reports demonstrated AR involvement in melanoma growth and invasion in vitro [44,76] and in vivo (Figure 1, right), eventually involving immune response blockage [59]. A recent work conducted on a small number of patients correlated AR-positive melanoma patients with worse survival when compared to those AR negative [58]. In this study, AR was shown to induce miR-539-3p expression that, targeting ubiquitin-specific peptidase 13 (USP13), abolished its de-ubiquitination activity on the microphthalmia-associated transcription factor (MITF) and determined the induction of the receptor tyrosine kinase AXL, with a consequent increase in metastases [58]. AR has been recently involved in other specific molecular pathways, for example its recruitment by the SRA-like long non-coding RNA (SLNCR) to the early growth response 1 (EGR1)-bound chromatin loci to repress p21 expression [77]. Furthermore, the activation of the non-genomic pathway, via the combination of the epidermal growth factor receptor (EGFR) and AR, enhanced AR activity itself and modified the melanoma-associated antigen protein-A11 (MAGE-A11), improving melanoma proliferation [28]. Another study described a decreased AR level in several tumors, including melanoma, when compared to the normal tissue counterparts, and with intratumoral receptor levels higher in males than in females. In contrast to most literature data on melanoma, high AR protein expression levels were associated with increased overall survival (OS) and progression-free survival (PFS) [78]. Recently, testosterone levels also gained some relevance as a possible cause of increased melanoma incidence in aging males. Multiple syngeneic metastatic mouse models demonstrated the importance of testosterone signaling on neutrophil maturation and function, since castration or androgen inhibition significantly increased melanoma burden [60].

## 5. Sex and Immunity

In the early stages of development, a high immunogenic phenotype characterizes melanomas, thus providing an ideal cancer model to understand the complex cross talk between tumors and immune cells and to give a possible explanation to sex differences in the host immune response. A scientific breakthrough in oncology research has been the recognition of immune system participation in the initiation, progression and, in some cases, the resolution of melanoma. Moreover, the recent introduction of the immune checkpoint inhibitors (ICI) in the therapeutic plan for melanoma patients has certified the crucial role of immunity in anticancer therapy, highlighting sex disparities [12,79,80,81]. Immunological sex differences concern both innate and adaptive immune responses and are influenced by either sex hormones or different specific genetic backgrounds between males and females [12]. In general, estrogens exert an immune-enhancing effect, contrasting to the immune-suppressive one of testosterone. Many different cell types express estrogen receptors (α and β ERs) (i.e., epithelial cells, lymphoid tissues and immune cells) that allow estrogen binding and signal activation [82,83,84]. Direct implication of estrogen-dependent effects on innate immunity have been recently evidenced in a syngeneic mouse model of melanoma where in vivo studies showed that the estrogen agonist erteberel, activating the ERβ signaling, was capable of augmenting innate immunity and suppressing lung metastatic colonization by recruitment of antitumor neutrophils to the metastatic niche [55]. On the contrary, castration in male mice led to an increased autoimmune response by the induction of the major histocompatibility complex II (MHCII) [85]. Moreover, estrogens enhanced the expression of MHCII on dendritic cells (DCs), while testosterone decreased it [86]. 

The E2/ERα axis plays a pivotal role in controlling functional responses of DC subgroups, and it is responsible for the epigenetic regulation in DC precursors, driving their differentiation also by modulating Interferon I (IFN-I) secretion [87]. In melanoma, DCs of tumor microenvironment act by modulating T cell activity and take part in the immune infiltration, which is considered an indicator of immune-therapy response [88]. In addition, a specific subgroup of DCs, named Langerhans’ cells and belonging to the skin immune system (SIS), is regulated by ERβ signaling [89,90]. Among the DC cells, the plasmacytoid DCs (pDCs) display major differences between women and men, their activity being under control of the E2/ERα axis and the X- linked Toll -like receptor 7 (TLR7) [91,92]. TLR7 belongs to the Toll-like receptor signaling and participates in the innate response against microbial infectious, favoring a better response in female association with a higher IFNα production [93]. The TLR signaling is an important autoregulatory mechanism that maintains tissue homeostasis, whose members are expressed on various skin cells, such as keratinocytes and melanocytes [94]. In particular, melanocytes express TLR 2-5 and TLR7, 9 and 10 [95]. Recent literature data evidenced their implication both in melanoma and non-melanoma skin cancer in supporting the immune escape [96,97,98]. Indeed, TLR agonists, targeting TLR7, 8, and 9, have been described as successful treatment options for melanoma and basal cell carcinoma (BCC), enhancing DC recruitment and T cell responses [99,100,101]. It is also important to note that several X chromosome genes take part in the innate immune function [102,103,104,105,106]. 

During the acute inflammatory response, the phagocytic activities of neutrophils and macrophages as well as the microbial killing by reactive oxygen species (ROS) are more efficient in females than in males. This female advantage has been also shown among melanoma patients. Melanoma cells exhibited significantly higher oxidative stress and produced larger amounts of ROS when compared to melanocytes and surrounding normal tissue [107]. ROS stimulated melanoma progression and metastatization through a number of changes, including (i) DNA modification, (ii) cell proliferation, (iii) tissue remodeling, (iv) immune surveillance escape, (v) pro-metastatic processes activation [108,109]. Malorni and colleagues demonstrated that males express lower levels of anti-oxidants, such as glutathione (GSH), catalase and superoxide dismutase (SOD) when compared to females, thus exhibiting a higher rate of oxidative stress [110,111]. Looking at the systemic influence of ROS on the metastatic phase of melanoma, a “ROS-sex issue” could be another reason for the differences eventually resulting in lower male survival rates [112,113,114]. 

In humans, natural killer cells (NKs) express both ERs and PRs, but not AR. Female hormones promote the induction of IFN γ, secretion of granzyme B and favor caspase-dependent apoptosis. In spite of these apparently contradictory data, male subjects exhibit a higher number of NK cells [12]. NKs participate in the first line of response against melanoma. One of the mechanisms used by melanoma for avoiding the CD8+ T cell antitumor action is the downregulation of MHC I. This reduction should support the removal of melanoma cells due to the capability of NKs to recognize and specifically eliminate cells expressing low levels of MHC I. Therefore, NKs appear a good target population for melanoma immune therapy [115,116,117]. 

The activation of adaptive immune responses has been demonstrated to counteract melanoma progression, metastatic spreading and therapy-related resistance. Two main different groups of melanoma-associated antigens have been characterized: (i) antigens expressed by normal and malignant melanocytes (as Gp100, tyrosinase, Melan-A and the isoform of tyrosinase-related protein, TRP-2 (INT2), and (ii) cancer testis antigens mainly expressed by transformed cells, such as melanoma antigen-1 (MAGE-A1), the highly immunogenic tumor antigen NY-ESO-1, and the preferentially expressed antigen of melanoma (PRAME) [118,119,120,121,122]. Female antigen-presenting cells (APCs) are more efficient than the male ones in both the presentation and initiation of a secondary response in primed lymphocytes. Indeed, androgen treatment of female mice reduced the efficiency of APC function, while estrogens exalted this function [123,124].

E2 concentration is considered the central rheostat for different adaptive immune response regulations: low E2 concentration induces Th1 response and cell mediated immunity, while high E2 concentration favors Th2 response and humoral immunity. 

Beyond the hormonal role, an additional explanation supporting sex differences in the immune system is the different genetic background, as females carry two X chromosomes and males just one. A mechanism to re-equilibrate gene expression is the random inactivation of one X copy in each female cell, thus making every woman a mosaic for X-linked expression [125]. An advantage of female mosaicism is the possibility to tolerate gene mutations responsible for X-linked diseases that severely affect males, such as those named X-linked primary immune deficiencies [126]. Furthermore, a percentage proximal to 15% of X-linked regions fails inactivation in women and, consequently, some genes in these regions might display a level of expression double than men [9]. It is important to highlight the presence of a high number of genes with immunological function on the X chromosome, possibly underlying not only the higher female immune response to infections, but also a positive effect on the anticancer immune responses. Among these genes, we can ascribe the Interleukin 2 Receptor Subunit Gamma (IL2Rγ) chain, the Interleukin 3 Receptor Subunit Alpha (IL3Rα) chain, and the Interleukin 13 Alpha (IL13α) chain, GATA-binding protein 1 (GATA1), Forkhead Box P3 (Foxp3) and CD40 Ligand (CD40L) [127]. On the contrary, this more reactive immune system, increasing female susceptibility to develop autoimmune disorders, represents a disadvantage [128,129].

Sex differences in lymphocyte subsets, including B cells, CD4+ and CD8+ T cells, have been demonstrated among adults. Females have a higher CD4+ T cell count and a higher CD4/CD8 ratio than age-matched males; whereas males have a higher CD8+ T cell frequency [130,131]. Studies in humans suggested a higher number of T regulatory cells (Treg) in healthy adult males compared to females, although some conflictual results regarding Treg frequency were reported [132]. 

Wesa and colleagues demonstrated that female melanoma patients have a high frequency of CD4+ TAA (tumor-associated antigen)-specific T cells compared to male patients, and that these cells are more prone to express an apoptotic phenotype in the presence of active disease [133]. The increase in and/or improved functions of tumor-specific T-helper (Th) cells could be a biological response to these differences due to it being involved in anti-tumoral responses [134]. 

**Figure 2:**
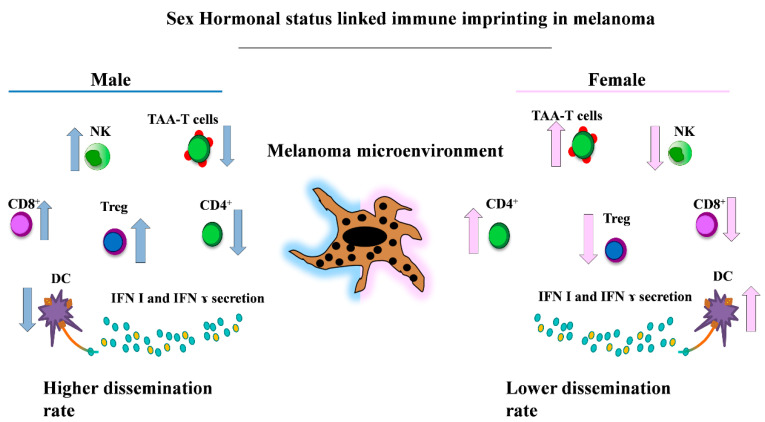
Key gender-related differences of the immune cell populations involved in melanoma. This representative picture shows the main immune cell populations present with higher frequencies in males (i.e., CD8+, Treg and NK cells [12,123,124,125]) (**left**) and in female patients (i.e., the immune phenotype enriched in TAA-T cells, CD4+ and DCs cells [80,123,124,126] with higher levels of circulating Interferon I (IFN I) and IFN γ [80,84,85]) (**right**). NK: natural killer; TAA: tumor-associated antigen; DCs: dendritic cells.

In recent years, in the immunological context of tumor biology, the immune checkpoint inhibitors emerged as promising therapeutic targets in different cancers, including melanoma [135]. The relationship between PD-1 and sex hormones recently emerged, despite the fact that data literature offered limited in vivo studies and conflicting results. Different data demonstrated that PD-1 is able to respond to sex steroids and that the hormone-mediated effect on PD-1 signaling might influence the regulation of autoimmune diseases [136]. In addition, estrogenic hormones were capable to modulate the programmed cell death ligand 1 (PD-L1) and B7-costimulatory molecules. Due to its contribution to immune evasion and induction of T regulatory cells, B7-H1 was associated with cells with pro-tumoral activity [137,138,139]. Lin and colleagues demonstrated that sexual hormones, in particular estrogens, modulated the Treg-linked B7-H1 immune suppression function. In a B7-H1 KO mouse model, female mice showed a better response to a B16 murine melanoma cell injection because a reduced Treg activation allowed a strong antitumor response when compared to males. Finally, in the same model, E2-mediated inhibition of the Treg function reduced primary tumor growth in female mice when compared to their male counterparts [140]. These results should induce a more careful estimation of sex differences in immune response and a sex-based interpretation of the therapeutic plans for anticancer immunotherapy (Figure 2).

## 6. Sex Differences and MiRNAs

The sex-linked differences affecting regulatory pathways in melanoma pathogenesis and the associated immune responses are further controlled by the emerging interaction between these functional signals and miRNA-specific epigenetic regulation. In these two decades of studies, microRNAs resulted as major post-transcriptional modulators of gene expression. In their mature form, miRNAs are 19–25 nucleotide long single strand RNAs, generally expressed in all cell types. A great number of works have described miRNA biogenesis and transcriptional regulation, and we refer to them for detailed explanation [141,142,143]. The high number of targets for a single miRNA and the capability of more than one miRNA to effectively repress the same gene result in complex and pleiotropic regulatory effects on cell physiology. MiRNAs regulate numerous cellular pathways and their alteration in pathological conditions determines a strong dysregulation of these pathways. In fact, in cancer pathogenesis, miRNAs can act as either oncosuppressors or oncogenes [144,145]. Of note, different tumors as well as diverse stages in the same cancer type display specific miRNA signatures [146]. In melanoma, specific expression profiles characterizing differences in disease progression, mutational state, as well as miRNA “facilitators” of drug resistance have been described [147].

Several studies, mainly in breast cancer, have demonstrated the direct correlation between miRNA expression and sexual hormones [148]. The miRNA-dependent regulation by direct targeting of ER mRNA at its 3’UTR and, vice versa, the capability of estrogen-specific pathways to modulate miRNA expression have been described and strictly depend on the specific activated receptors [149,150,151]. In melanoma, the effect of E2 treatment on miRNA expression and, more in general, their real functional link remain essentially unknown. Nonetheless, the elucidation of miRNA and ER-functional networks might help to understand some controversial aspects of female survival advantage compared to male survival in initial phases of melanoma development, or the reduction in these advantages in postmenopausal women when female estrogenic concentration vertically declines [32,34]. It is here important to consider that melanoma could be classified among the hormone-sensitive tumors according to complex overlapping actions played by estrogens and androgens, particularly by the opposite effects of α and β estrogen receptors (ER) [28]. Some functional parallelisms with miRNAs controlled by the estrogenic action in breast cancer might help to understand if some miRNA alterations in melanoma might be under hormonal control. An important example is the feedback loop involving the miR-221 and -222 cluster and ERα in breast cancer. A regulatory circuitry exists on one side based on the direct interaction of ERα with the estrogen receptor-binding site in the promoter region of miR-221&222 to induce their expression [152], on the other side on miR-221&222 direct targeting of ERα. Indeed, these two miRNAs and ERα are negatively related [153].

In breast cancer, miR-221 and -222 overexpression, and in turn ERα reduction, were shown to trigger cancer cell proliferation and invasion [154]. Furthermore, the miR-221 and -222-dependent decreased expression of ERα reduced cell sensitivity to the tamoxifen endocrine therapy, favoring resistance and eventually exacerbating malignant progression of disease. Accordingly, xenograft tumors treated with a specific antagomir, the down-modulating of miR-221 and -222 expression removed this effect [155]. Although the possible estrogenic regulation of miR-221 and- 222 expression in melanoma progression remains to be defined, the roles and regulation of these two miRNAs have been deeply studied. Similarly, to breast cancer, melanoma progression and spread require miR-221 and -222 expression, while ERα expression is lost. At least six target genes were revealed, including p27^Kip1^, tyrosine-protein kinase Kit (c-KIT), ETS proto-oncogene 1 (ETS-1), AP-1 transcription factor subunit (c-FOS), Activating enhancer binding Protein 2 α (AP2α) and Stearoyl-CoA Desaturase 5 (SCD5), all with direct or indirect tumor suppressor roles according to the central roles of miR-221 and -222 in melanoma proliferation and dissemination [156,157,158,159,160]. 

**Figure 3:**
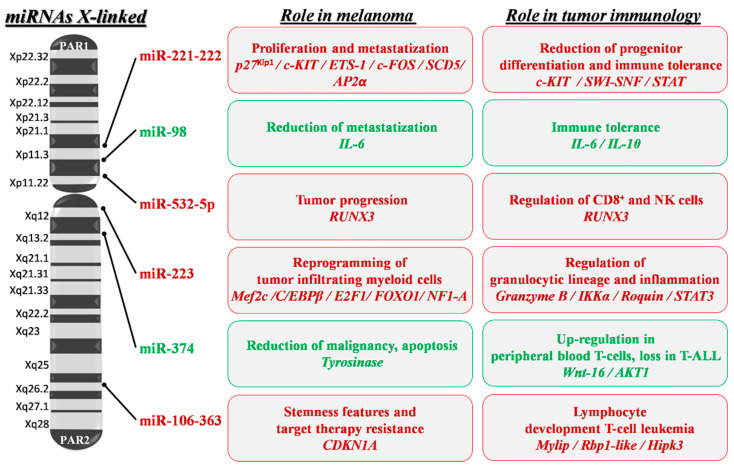
X-linked miRNA mapping and key roles in melanoma development and progression. Schematic depiction of miRNA localization on the human X chromosome: miRNAs with a confirmed role in melanoma pathogenesis are shown. Red writing inside boxes indicate miRNAs, target genes (italics) and their downstream oncogenic effects; green writing indicates those with oncosuppressive functions. PAR1 and 2: pseudoautosomal region 1 and 2; p27^Kip1^: cyclin-dependent kinase inhibitor 1B; ETS-1: ETS proto-oncogene 1; SCD5: Stearoyl-CoA Desaturase 5; SWI-SNF: Switch/Sucrose Non-Fermentable; STAT: Signal Transducer and Activator of Transcription; IL-6 and 10: Interleukin 6 and 10; RUNX3: Runt-related transcription factor 3; IKKα: Inhibitory-KB Kinase α; STAT3: Signal Transducer and Activator of Transcription 3; MEF2C: Myocyte Enhancer Factor 2C; C/EBPβ: CCAAT/enhancer-Binding Protein β; E2F1: E2F transcription factor 1; FOXO1: Forkhead Box protein O1; NF1-A: Nuclear Factor 1 A; Wnt-16: Wnt family member 16; AKT1: AKT serine/threonine kinase 1; CDKN1A: Cyclin-Dependent Kinase Inhibitor 1A; Rbp1-like: Retinol Binding Protein 1-like; Hipk3: Homeodomain-Interacting Protein Kinase 3.

Thanks to the high number of miRNAs present on the X chromosome (approximately 120 miRNAs) compared to both autosomes and mainly to the Y chromosome (at present only 4 miRNAs), X-linked miRNAs might have a role in sex differences evocated in melanoma immune response. Potentially, miRNAs might escape dosage compensation in association with the genomic co-localized genes evading X inactivation [161,162]. Indeed, a sex different susceptibility to cancer development concerns different solid tumors, with males in some cases at higher risk compared to females [163,164,165]. Several miRNAs involved in hematopoietic lineage differentiation and in pathological conditions play a role in chronic inflammation as a predisposing factor in the onset and progression of cancer [166,167]. Thus, it might be relevant to consider the possible functional interconnections between X-linked miRNAs and immune responses underlying sex differences in melanoma (see Figure 3).

Once more, miR-221 and -222 are the most studied X-located oncomiRs that result in strongly deregulated different forms of cancer, including breast cancer, prostate cancer, liver cancer, bladder cancer, thyroid cancer, glioblastoma and melanoma [168]. Their important role and sex-related modulation was assessed in cardiovascular diseases where miR-222 indirectly reduced the endothelial nitric oxide synthase formation through ETS-1 targeting in cardiac vascular cells [169]. The ETS-1-miR-222 circuitry was also associated with melanoma progression, miR-222 induction being strictly dependent on the constitutive ETS-1 phosphorylation and compartmentalization in metastatic tumors and ETS1 directly targeted by miR-222 [157]. In hematopoiesis, miR-221 and -222 were shown to reduce differentiation of the embryonic hematopoietic progenitor cells because of their direct targeting of c-KIT mRNA [170]. We imagine that increased miR-221 and -222 levels, representing either melanoma cell-intrinsic or immune system-extrinsic factors, can contribute via exosome release to disease progression [171,172,173]. 

The miR-17-92 cluster includes 15 miRNAs organized in three paralogue groups on different chromosomes. Among them, the miR-106-363 paralogues are localized on the X chromosome and include six miRNAs (miR-106a,-18b,-20b,-19b2,-92-2 and -363). Due to sequence similarity, genomic organization and functional connections, these miRNAs are further organized in a different family, named miR-17,-18,-19 and -92. All were designated with oncogenic potential in different forms of cancer [174]. Mainly, the miR-17 family was linked to melanoma progression and individuated in non-responders or patients expressing high levels of PD-L1 and resistance to BRAF/ MEK inhibitors [147,175]. In addition, miR-106a and miR-363 play a role in the immune regulation by controlling lymphocyte development. When overexpressed, these miRNAs act as oncogenes and are strongly implicated in T cell leukemia development by targeting myosin regulatory light chain-interacting protein (Mylip), retinoblastoma-binding protein 1-like (Rbp1-like), and homeodomain-interacting protein kinase 3 (Hipk3) [176]. In melanoma, miR-363-3p was indicated in the targeting of the inhibitor of cell cycle progression of cyclin-dependent kinase inhibitor 1A (CDKN1A). This targeting was associated with the parallel expression of Hif-1α and the acquisition of stemness features on melanoma cells as specified by CD133, CD271, Jarid1B, and Nanog expression [177].

Tumor-infiltrating myeloid cells are immature tumor-infiltrating cells of hematopoietic origin that favor cancer progression by a mechanism of immune suppression described in the breast and in melanoma. The myeloid-derived suppressive cells were also crucial for the metastatic niche formation, inducing expression of factors with an invasive-promoting action. Different X-linked miRNAs have been described to have a role in this suppressive mechanism. The more studied is miR-223, which when overexpressed, reduced maturation of the granulocytic cell lineage that regulates the Mef2c transcription factor necessary for myeloid progenitor proliferation, and it acted as negative modulator of inflammatory responses in animal models [178,179]. MiR-223 is highly expressed in melanoma. In a mouse model of melanoma metastasis, miR-223 in association with miR-21, miR-29a and miR-142-3p, was demonstrated to support the reprogramming of tumor-infiltrating myeloid cells and the acquisition of pro-metastatic and angiogenic properties [180].Validated targets for miR-223, during inflammation, include granzyme B, Inhibitory-KB Kinase α (IKKα), Roquin and Signal Transducer and Activator of Transcription 3 (STAT3), while cancer-associated targets include CCAAT/enhancer-Binding Protein β (C/EBPβ), E2F transcription factor 1 (E2F1), Forkhead Box I A (FOXO1) and Nuclear Factor I A (NFI-A) [181].

MiR-532-5p is upregulated in primary and metastatic melanoma cell lines and in tumor samples. This upregulation was associated with down-modulation of its target Runt-related transcription factor 3 (RUNX3), a member of the runt-related family of genes that are known as developmental transcriptional regulators. These genes are important in the progression of a variety of human cancers and are involved in the differentiation program of normal tissues [182]. It is interesting to note that RUNX proteins regulated the transcription of the MDR1 gene in cytotoxic CD8+ lymphocytes and NK cells. The MDR1 protein was required for the accumulation of cytotoxic T lymphocytes (CTL) cells after acute viral infections or in the protective function of memory-T cells following microbial challenge. In view of this new research, the counterproductive effect of the MDR1 inhibition as an anticancer therapeutic strategy was suggested. In this new context, miR-532-5p-dependent epigenetic regulation might be co-responsible for the reduction of tumor-infiltrating CD8+ cells in the tumor microenvironment [183]. 

Among other X-linked miRNAs, miR-374, part of the human miR-374 family, down-regulates tyrosinase expression and reduces melanoma malignancy by attenuation of WNT signaling, thus promoting melanoma cell apoptosis in a mouse model of melanoma [184]. In a combined expression profile, the miR-374 family was found upregulated in monocytes and granulocytes vs. progenitor cells, while in the immune context, miR-374b was up-regulated in peripheral blood where T cells targeted Wnt-16 and AKT1 [185,186]. Finally, several X-linked miRNAs contributing to the melanoma-associated immune regulation act by modulating the Toll-like receptor-mediated responses. Indeed, the development of agonists for the TLR signal has been evocated to trigger new therapeutic strategies for the induction of anticancer immune response [187]. An interesting example is miR-98. This miRNA is down-modulated in melanoma progression where it plays a negative feedback loop with the pro-inflammatory IL6 [188], but it also acts as a regulator of IL10 expression in response to microbial infection or after lipopolysaccharide (LPS) stimulation [189]. Furthermore, miR-221 and -222 oncomiRs, besides a number of other functions, participate in the regulation of TLR response-inducing tolerance to microbial infection essentially through TNFα (Tumor Necrosis Factor α) degradation by regulation of Switch/Sucrose Non-Fermentable (SWI/SNF) and Signal Transducer and Activator of Transcription (STAT) [190]. It is important to underline the parallelism between TLR and miR-221 and -222 functions whose expressions might contribute to the increased immune impairment evidenced in melanoma patients eventually favoring tumor metastatization.

## 7. Sex Differences and Response to Therapies

Starting from Clark’s observation in 1969, melanoma seemed less malignant in women than in men [191]. Since then, many other large studies confirmed this result, reporting a 20–30% female advantage worldwide. A recent study based on a patient cohort of approximately 16.000 stage II–IV cutaneous melanoma patients confirmed the female advantage in melanoma survival by using the latest prognostic staging system for risk stratification [192]. 

Moving forward, in regard to the relevance of sex and gender in clinical research, we should start from considering the underrepresentation of women in clinical trials. At present, despite many invitations for inclusion of a balanced number of men and women, no significant improvements were obtained and women, particularly in the initial phases of the studies, still represent 20–25% of the total [193]. 

In addition, even when a significant number of women are enrolled, often data are not properly stratified for the analyses [194,195]. In accordance with the sex and gender equity in research (SAGER) guidelines, differences between men and women should be always considered in the evaluation of response to therapies, toxicities and outcomes [196]. More important, in Italy, the Law 3/2018, particularly article 1, specifically includes the concept of gender medicine in clinical trials for human medicinal products, highlighting the importance of a gender-specific approach [197]. On this basis, we should expect to see some gradual progress in the near future, starting from the point that that men and women are not the same [198].

In the last few years, melanoma therapeutic approaches showed a rapid evolution with the main goal of increasing clinical efficacy and the duration of responses. The first promising result was obtained by targeted therapy against the activating mutations of BRAF, present in 40–60% of cutaneous melanomas with a similar distribution among men and women [199,200]. Major benefits were achieved with the combination of BRAF and MEK inhibitors in order to avoid the so-called “paradoxical activation” of the MAPK pathway downstream to BRAF [201,202]. More recently, important results were obtained through the inhibition of some molecules involved in T cell suppression. In particular, the impairment of CTLA-4, PD-1 or PDL-1 functions through the immune checkpoint inhibitors (ICIs) gave impressive responses in several types of cancers, including metastatic melanoma [203]. Although some sex differences were observed in the efficacy of more traditional therapies [204], according to the known disparities in the immune system with women sustaining stronger responses [12], it was reasonable to expect diverse sex-associated anti-tumor effects of ICIs [205]. In fact, in the initial studies in proper in vivo models, immune checkpoint inhibitors were more effective in female mice than in their male counterparts [140]. Conversely, in human clinical trials, unexpected better outcomes were shown in men. Several meta-analyses evidenced this result in cancer-randomized clinical trials treated with ICIs, initially suggesting a more evident benefit associated with the anti-CTLA4 treatment for males vs. females [206]. Looking for a higher statistical power, an elevated number of patients (>11,000) was analyzed as part of 20 randomized controlled trials based either on CTLA-4 or PD-1 inhibitors. Results confirmed and extended the increased efficacy of immunotherapy in men vs. women [80]. Many hypotheses can be proposed to explain these disparities, including the sex-related differences of the immune system at baseline conditions, as indicated by the higher number of CD8+ T lymphocytes and the lower CD4+/CD8+ ratio in males than in females [12]. In addition, the higher amount of Treg cells in men, the subpopulation preferentially depleted by anti-CTLA-4 antibodies, could play a role in the different responses, subsequently producing a more significant male benefit [207]. 

It is important to note that the results associated with ICI plus chemotherapy appear different, giving more benefits to women than to men. This might possibly depend on the chemotherapy-derived increase in the mutational load of tumors, their antigenicity and the stronger immune response of women [10]. The complexity is further augmented by disparities between the ICI agents, for example, the anti-CTLA4 antibodies being more sex-related than the anti-PD-1 ones. Indeed, sex-related differences seem more relevant to overall survival in melanoma patients than in other cancers [208,209].

Another intriguing result reported the association between obesity and outcomes in male metastatic melanoma patients treated with targeted or immunotherapies. In men, a high BMI was associated with a doubled survival time, whereas this result was not detected in women. One explanation might be that obesity in men results in higher levels of estrogens due to aromatase activity converting androgens into estrogens [210]. A role might be also played by the sex hormone-binding globulin (SHBG), a glycoprotein acting as a transporter of testosterone and estradiol in plasma. SHBG is inversely associated with obesity, displaying gender-associated differences. Indeed, women have higher levels of SHBG when compared to men, and SHBG amounts are further reduced in obese men [211].

To conclude, it is important to note that even in adverse events, some sex-related differences can be observed. Generally, women have more adverse reactions to chemotherapy than men, which might derive from differences in the pharmacokinetics and pharmacodynamics of the drugs [212]. Furthermore, worst might be the expected risks of severe symptomatic adverse effects experienced by women after immunotherapy due to their stronger immune responses [213,214]. Nonetheless, because of the short follow up of immunotherapy, some discrepancy still exist in regard to this point [215]. 

Altogether, these data strongly support the relevance of balanced enrolment of men and women in clinical trials in order to consistently confirm differences, evaluating disparities between populations, ages, and sites [216]. Sex and/or gender represent a variable to consider in order to obtain impartial approaches, selecting the best therapeutic option with the lowest adverse effect for each person. 

## 8. Conclusions

The increment of data on the pathogenic mechanisms made melanoma finally attackable also in its metastatic phase by target therapy and, more recently, by immunotherapy. However, many shadows persist that need to be dissipated. Among them, the mechanisms underlying sex and/or gender differences represent a key point requiring further studies. Epidemiological data confirm female advantages in both incidence and mortality, and these differences are present in all the analyzed racial and ethnic groups. Although disparities can be partly ascribed to different gender-related behaviors, recent results indicate biological variations, including genetic and epigenetic aspects, as key players. 

An important action might depend on X chromosome regulation. The random inactivation of one of the two X chromosomes in women, together with the possibility of some X-regions escaping this regulation, surely contribute to female benefits. In addition, the X chromosome is enriched with both coding and regulatory non coding genes, - such as miRNAs, playing immunological functions. The complex crosstalk among different cells of the tumor microenvironment and the influence of the hormone regulatory signals on these cells might affect immunotherapy response, with a profound impact on results. Therefore, evaluating gender variance and connecting these aspects will improve patient stratification and assist in tailoring the provided healthcare to the individual patient.
